# Natural polymorphisms and unusual mutations in HIV-1 protease with potential antiretroviral resistance: a bioinformatic analysis

**DOI:** 10.1186/1471-2105-15-72

**Published:** 2014-03-15

**Authors:** Carlos Mata-Munguía, Martha Escoto-Delgadillo, Blanca Torres-Mendoza, Mario Flores-Soto, Mildred Vázquez-Torres, Francisco Gálvez-Gastelum, Arturo Viniegra-Osorio, Marcelo Castillero-Manzano, Eduardo Vázquez-Valls

**Affiliations:** 1 Doctorado en Farmacología, Departamento de Fisiología, Centro Universitario de Ciencias de la Salud, Universidad de Guadalajara, Guadalajara 44340, México; 2 Laboratorio de Inmunodeficiencias y Retrovirus Humanos, Centro de Investigación Biomédica de Occidente, CMNO, IMSS, Guadalajara 44340, México; 3 Unidad de Investigación Cardiovascular, Departamento de Fisiología, Centro Universitario de Ciencias de la Salud, Universidad de Guadalajara, Guadalajara 44340, México; 4 División de Excelencia Clínica, Coordinación Médica de Unidades de Alta Especialidad, Unidad de Atención Médica, IMSS, México, D.F 06700, México; 5 UMAE, Hospital de Especialidades, CMNO, IMSS, Guadalajara 44340, México; 6 Departamento de Producción Agrícola, Centro Universitario de Ciencias Biológicas y Agropecuarias, Universidad de Guadalajara, Zapopan 45110, México; 7 Departamento de Clínicas Médicas, Centro Universitario de Ciencias de la Salud, Universidad de Guadalajara, Guadalajara 44340, México; 8 Departamento de Farmacobiología, CUCEI, Universidad de Guadalajara, Guadalajara 44430, México

**Keywords:** Antiretroviral agents, Bioinformatics, Molecular docking simulation, Drug resistance, HIV protease, *In silico*, Polymorphism, Mutations

## Abstract

**Background:**

The correlations of genotypic and phenotypic tests with treatment, clinical history and the significance of mutations in viruses of HIV-infected patients are used to establish resistance mutations to protease inhibitors (PIs). Emerging mutations in human immunodeficiency virus type 1 (HIV-1) protease confer resistance to PIs by inducing structural changes at the ligand interaction site. The aim of this study was to establish an *in silico* structural relationship between natural HIV-1 polymorphisms and unusual HIV-1 mutations that confer resistance to PIs.

**Results:**

Protease sequences isolated from 151 Mexican HIV-1 patients that were naïve to, or subjected to antiretroviral therapy, were examined. We identified 41 unrelated resistance mutations with a prevalence greater than 1%. Among these mutations, nine exhibited positive selection, three were natural polymorphisms (*L63S/V/H*) in a codon associated with drug resistance, and six were unusual mutations (*L5F, D29V, L63R/G, P79L* and *T91V*). The *D29V* mutation, with a prevalence of 1.32% in the studied population, was only found in patients treated with antiretroviral drugs. Using *in silico* modelling, we observed that D29V formed unstable protease complexes when were docked with lopinavir, saquinavir, darunavir, tipranavir, indinavir and atazanavir.

**Conclusions:**

The structural correlation of natural polymorphisms and unusual mutations with drug resistance is useful for the identification of HIV-1 variants with potential resistance to PIs. The D29V mutation likely confers a selection advantage in viruses; however, *in silico*, presence of this mutation results in unstable enzyme/PI complexes, that possibly induce resistance to PIs.

## Background

Diversity of viral populations is a result of sophisticated recombination, replication and/or selection events that induce drug-resistant human immunodeficiency virus type 1 (HIV-1) variants. The lack of reverse transcription corrections, transitional printing and transversion mutations, along with viral recombination, has resulted in the emergence of HIV-1 variants with high resistance to pharmacological stressors [1,2]. These variants form populations that evade antiretroviral agents, due to emerging phenotypic changes within and around the active enzyme site [3]. These mutations, which give rise to drug resistance, result in reduced efficacy of highly active antiretroviral therapy (HAART) [4]. Correlations between genotypic and phenotypic tests with treatment, clinical history, and significance of mutations identified in HIV-1 of infected patients are used to determine the presence of mutations that confer resistance to protease inhibitors (PIs) [1].

Disruption at interaction sites causes an alteration in affinity between proteins and their inhibitors, and has been recognized as a property of drug resistant HIV-1 proteins [5,6]. Protein folding simulation models can create Local Elementary Structures (LES). These secondary structures are stabilized by amino acids that interact with the polypeptide chain [7]. Using the Gromacs software (version 3.0), LES were found to form in protease (PR) regions 23–33, 74–78, and 83–92, and also docked in a folding nucleus [8]. Other studies have shown that mutations further from the active site can alter the flexibility of HIV-1 PR, inducing structural changes that affect the efficacy of most PIs currently used [9]. Theoretical studies, either alone or in combination with experimental methods, have pointed to an increase in the flexibility of mutant enzymes at various sites, including the active site, as a resistance mechanism that causes a decrease in the affinity of PIs [10]. Part of the cause of such flexibility could be the unusual mutations that generally emerge only after "major" and "minor" resistance mutations have been introduced [11]. Other mutations that can affect the interaction between PR and PIs are natural polymorphisms and unusual mutations in positions that confer drug resistance. Although the main mutations associated with drug resistance have been characterized [12,13], little is known about the influence of natural polymorphisms and unusual mutations with respect to the development of drug resistance. The aim of this study was to describe an *in silico* experiment that showed structural correlations between natural HIV-1 polymorphisms and unusual HIV-1mutations in the PR region of HIV-1 *pol* with potential PIs resistance.

## Methods

### Sequence data

We analysed 151 HIV-1 sequences from Mexican patients who had been tested for resistance to antiretroviral drugs between 2005 and 2011 in the Laboratory of Immunodeficiencies and Human Retroviruses, Western Biomedical Research Center, Mexican Institute of Social Security. Sequences were obtained from 22 naïve, and 129 treated patients that were not responsive to drugs. Sequences were registered in the GenBank database [14], with the following accession numbers: [EU045452–EU045489; GU382757–GU382851; GU437199–GU437200; and KC416212–KC416227]. All sequences were analysed for the presence or absence of highly mutated sequences using HYPERMUT software (version 2.0) [15]. For a reference sequence, we used the subtype B consensus sequence, which was derived from an alignment of subtype B sequences maintained at the Los Alamos HIV Sequence Database (LANL), and available from the HIV Drug Resistance Database (HIVDB), Stanford University [16].

### Phylogenetic analysis

Nucleotide homology analysis for HIV-1 sequences was conducted using the NCBI Genotyping Tool program [17]. Subtype determinations were further confirmed by phylogenetic analysis performed with the Molecular Evolution Genetics Analysis (MEGA) software package (version 5.0) [18], which includes the recommended reference sequence sets, available from the Los Alamos HIV Sequence Database [19]. Prior to all phylogenetic analyses, HIV-1 *pol* sequences were aligned using Clustal X (European Bioinformatics Institute, EMBL) [20]. Sequences with 100% homology were excluded from the analysis. The nucleotide distance matrix was generated using the Kimura two-parameter Neighbour-joining method [21]. The statistical robustness of the generated trees was verified by bootstrap analysis of 1000 replicates.

### Detection of multidrug resistance phenotypes in HIV-1 protease

The genetic changes associated with drug resistance in viral sequences were established according to HIVdb algorithm version 6.0.9 (http://hivdb.stanford.edu) [22]. The interpretation of drug resistance was performed at various levels of susceptibility for the following USA Food and Drug Administration (FDA)-approved PIs: atazanavir (ATV); darunavir (DRV); amprenavir (APV); indinavir (IDV); lopinavir (LPV); saquinavir (SQV); tipranavir (TPV); nelfinavir (NFV);and ritonavir (RTV). The resistance mutations were classified as major or minor according to HIVdb criteria, or as natural polymorphisms or unusual mutations if they were not associated with resistance [16]. The prevalence (*p*) for each mutation in the protease region of *pol* was quantitatively determined as the frequency of the mutation (*M*) among total sequences evaluated for each position (*N*), *p = M/N*, using Microsoft Excel 2010. The genetic variation was calculated as the total number of mutations at a nucleotide position divided by the number of evaluated sequences. The Phenotypic Variation (PV) was defined as the percentage (%) of amino acid substitutions for each position relative to the consensus sequence. For each region, the PV was classified as follows: conserved, <1%; semi-conserved, 1 to <5%; variable, 5 to <10%; and highly variable, ≥10%. Values found below the 15th percentile and above the 75th percentile were not considered. Phenotypic mutations with a prevalence of ≥1.0% among 151 amino acid sequences were compared for each PI against the IAS–USA drug resistance mutations list [12]. The structural conservation of PR was defined in a complementary way to that of PV.

### Analysis of selective pressure

The selective pressure and reconstruction of the ancestral state for each PR codon was determined using a maximum likelihood (ML) substitution model and the HyPhy algorithm, included in MEGA5 package [23,24]. The synonymous site divergence (dS) and nonsynonymous site divergence (dN) per branch was estimated using the Muse–Gaut codon model [25]. The values of the ML model were estimated from the topology of the phylogenetic tree. The probability of rejecting the hypothesis of neutral evolution was significant with *p* ≤ 0.05. The standardized values of dN–dS were obtained by the total number of substitutions in the tree (calculated as substitutions expected by site). To distinguish between drug pressure and immune system pressure, results were compared using the HIV positive selection mutation database [26].

### Molecular modelling

Once the natural polymorphisms and unusual mutation codons with positive selection (dN/dS > 1) and prevalence >1% were obtained, homology modelling was used to predict changes in the PR structure. Homology modelling of natural polymorphisms and unusual mutations followed these steps: (i) template selection; (ii) structural alignment; (iii) model construction; and (iv) refinement [27]. To select the template, HIV-1 protease X-ray crystal structure FASTA sequences available from the Protein Data Bank (PDB) [28] and the HIV-1 subtype B consensus sequence available from the HIVdb were aligned using ClustalX [20]. The PR sequence exhibiting greatest identity with HIV-1 subtype B consensus (wild-type template) was chosen as the template for modelling mutant proteases (PRs). The resistant PRs used for reference were modelled with each major PI resistance mutation. Every mutant protein was modelled using a Swiss-Model workspace, which showed the identity (%). The expected alignment value with the template sequence (E) and the Qualitative Model Energy Analysis (QMEAN4), which estimates the absolute quality model, ranged from 0–1 [28,29].

### Estimation of the free energy of binding

Using the Autodock/Vina application on a LINUX platform, which had the PyMOL (version 1.4.1) molecular graphics system installed, we estimated the free energy of binding of the complex between mutant PR structures and Pls [30]. Rectangular boxes were used to define the binding sites and these were adjusted by providing specific coordinates of active PR sites before each docking.

Receptor and ligand representations in the Protein Data Bank, Partial Charge & Atom Type formats (pdbqt) containing atomic charges, atom type definitions and topological information, were produced using Autodock/Vina [30]. To determine if the differences caused by natural polymorphisms and unusual mutations had any effect on the free energy of binding of Pls, the free energy values obtained for the resistant protease/ligand complexes were compared. Natural polymorphisms or unusual mutations with lower or equal affinity to PIs compared with reference proteins containing drug resistance mutations indicate positive resistance. Higher affinity was considered to favour susceptibility of the HIV-1 variant to PIs. The coupled proteases included the wild-type PR [PDB: 1GNO], PRs with major drug-resistance mutations and PRs with natural polymorphisms or unusual mutations at codons having positive selection.

### Measurement of distances between protease residues and PIs

To evaluate the natural polymorphism and unusual mutation atoms that affect the affinity of PIs, we measured the distances (Å) between the amino acid residue C_α_-atoms implicated in drug resistance, and the closest heteroatoms of the PIs. Complexes that showed signs of free energy of binding were analysed, suggesting increased drug resistance because of the presence of natural polymorphisms and unusual mutations. Distances were compared with those obtained for the same pair of atoms in the wild-type and resistant PR structures available from the PDB [28]. All interatomic distances were measured with PyMOL (version 1.4.1) [31].

## Results and discussion

### Genetic relationships of HIV-1 variants isolated from Mexican patients

Phylogenetic analysis of the 151 HIV-1 protease fragment nucleotide sequences was conducted using a Neighbour-joining tree. Phylogenetic relationships were grouped into the internal nodes of the tree, using subtype B reference sequences [GenBank: U63632 and U21135]. The HIV-1 variants isolated from Mexican patients, and confirmed by analysis with the NCBI Genotyping Tool, were subtype B. This result is consistent with other molecular epidemiology studies of Mexican HIV-1 patients, with or without antiretroviral intervention, where subtype B prevails [32,33].

### Drug resistant phenotypes and genotypes of HIV-1 protease

**Figure 1:**
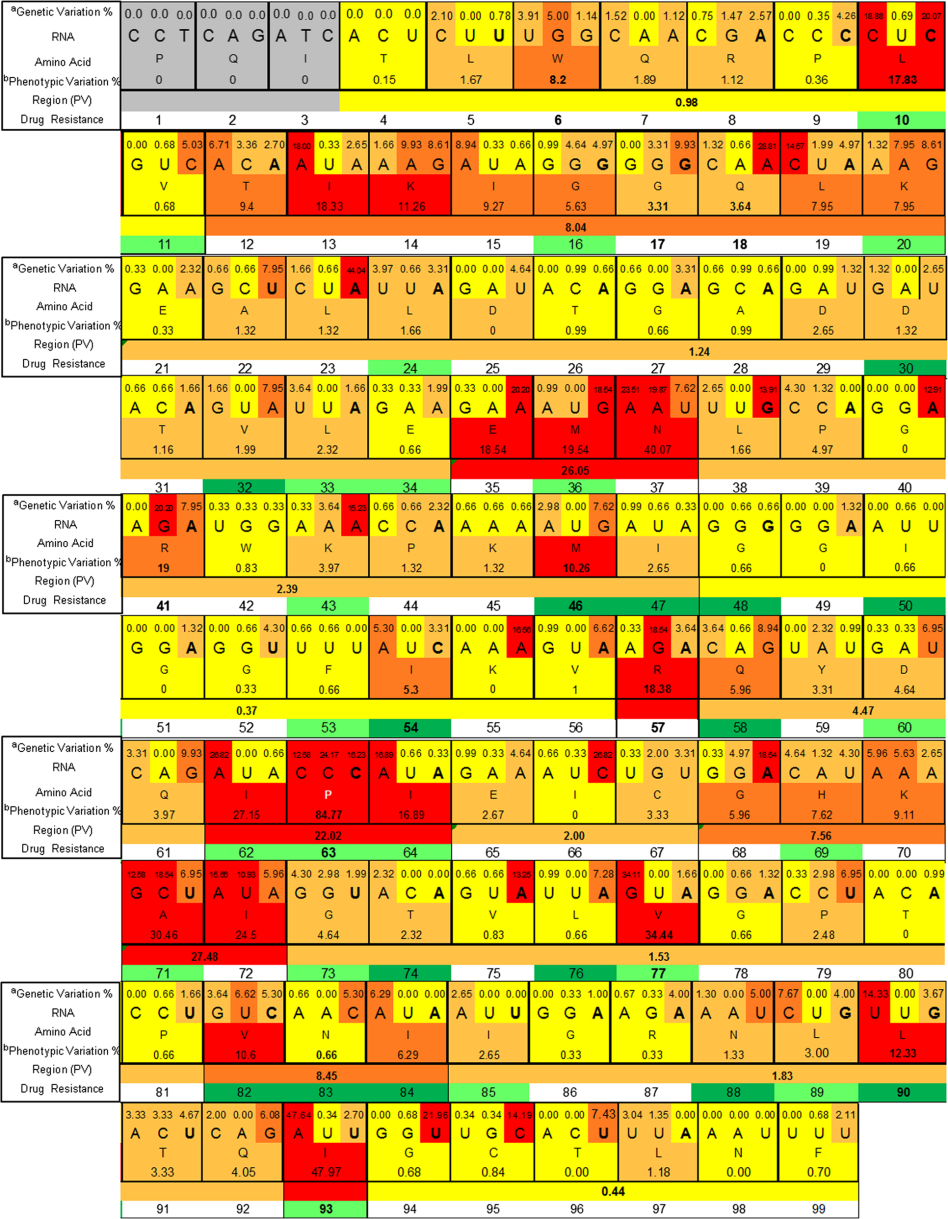
**Genetic and phenotypic representation of primary structure variation within the HIV-1 protease consensus.** Codons 1–3 are shown in grey and were not included in our analysis. Conserved regions are shown in yellow, semi-conserved regions in ochre, variable regions in orange and highly variable regions in red. The major (dark green) and minor (light green) resistance mutations are indicated for each codon. ^a^Genetic variation = total number of mutations at the nucleotide position/number of sequences evaluated. ^b^Phenotypic variation = total number of mutations at the amino acid position/number of sequences evaluated.

**Figure 2:**
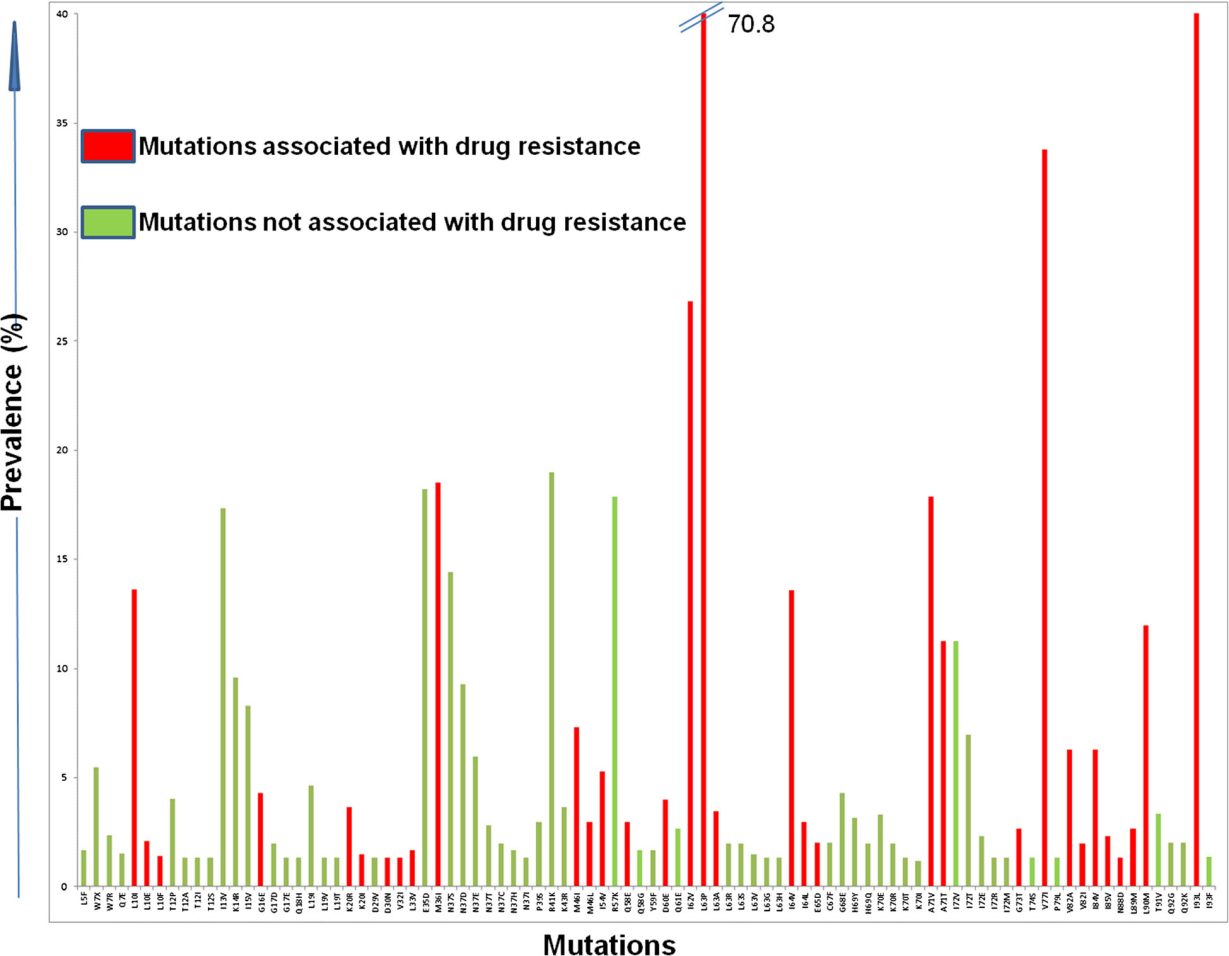
**Prevalence of mutations within HIV-1 PR *pol*.** Red bars represent mutations associated with drug resistance, and the green bars represent natural polymorphisms and unusual mutations not associated with drug resistance.

Non-synonymous genetic changes largely contribute to phenotypic changes [34]. Because of degeneracy in the genetic code, transcription and translation errors during the viral replication cycle, along with functional, structural, pharmacological and immunological selection pressures, there is no absolute mathematical relationship between genetic and phenotypic variations [35]. Variations in the primary structure of the 151 PR sequences are presented in Figure 1. Of the 36 codons associated with major or minor resistance, 19 showed PV <5% (L11, L24, D30, V32, L33, E34, K43, I47, G48, I50, F53, D60, G73, T74, L76, N83, I85, N88 and L89). Among these codons, G48, I50 and F53 were present in conserved regions, and L24, D30, L76 and N88 in semi-conserved regions. Six codons (G16, K20, I54, Q58, H69 and I84) displayed PV of 5–10%. Of these, only codons I54 and Q58 were located in conserved and semi-conserved regions, respectively. The remaining 11 codons associated with drug resistance (L10, M36, M46, I62, L63, I64, A71, V77, V82, L90 and I93) had variation >10%, with L10 and I93 present in conserved regions, and M46, V77 and L90 in semi-conserved regions. There was also a variable PV of 5–10%, in the codons neighbouring the drug resistance positions (T12, I15, L19, G68, and K70), with codons E35, N37, R41, R57, and I72 highly variable. Figure 2 illustrates the mutations with prevalence ≥1% found in the protease region of HIV-1 that were present in the 151 PR sequences examined. According to the IAS-USA, the mutations associated with drug resistance, with a *p >*10%, were *L10I*, *M36I*, *I62V*, *L63P*, *I64V*, *A71V/T*, *V77I*, *L90M,* and *I93L*[12,36,37].

Structural studies of PRs have reported a slight widening of the active site due to mutations associated with drug resistance for the majority of PIs [9,10,38]. However for other inhibitors, such as IDV which is characterized by three aromatic rings, structural changes are caused by mutations at the active site and adjacent positions [39].

### Prevalence of natural polymorphisms and unusual mutations in PRs without established drug resistance

Table 1 shows the natural polymorphisms or unusual mutations with a *p >*1% that were found in the PR sequences of HIV-1 isolated from the Mexican patients. These are weakly associated with PI resistance, but are not included in the IAS–USA guides or the HIVdb as accessory or minor mutations [16,40,41]. Of the 14 mutations, only *L63A* and *H69Y* were found in drug resistance positions, and *T12A/I*, *I15V*, *E35D*, *N37D/E*, *R57K*, *K70E* and *I72V* were contiguous to positions associated with resistance. Overall, these mutations have little effect on drug susceptibility; however, a phenotypic change in any of them could have relevance to the affinity to one or more PIs [6,42]. These mutations, in combination with resistance mutations, might have an effect on the dynamics of the evolution of cross-resistance [43].

**Table 1:** **Polymorphisms or unusual mutations (*p*** **> 1%) weakly associated with PI resistance in HIV-1 protease from treated and naïve individuals according to the HIVdb**

**Mutation**	***p *** **(%)**	**PV (%)**	**Region (PV)**	**Association with drug resistance**	**Classification**
*W6R*	2.34	8.20	C (0.98)	Found in indinavir-resistant PR [44]	UM
*T12A/I*	1.34/1.34	9.40	V (8.04)	*T12A* decreased in patients treated with PIs. [45]*T12I* appears in cell culture in the presence of SQV [46]	NP/NP
*I13V*	17.33	18.33	V (8.04)	Found in isolates from patients treated with NFV [47]	NP
*I15V*	8.28	9.27	V (8.04)	Associated with reduced virological response to RTV + SQV therapy [48]	NP
*E35D*	18.21	18.54	HV(26.05)	Associated with reduced *in vivo* virological responses to RTV/AMP [49]	NP
*N37D/E*	9.27/5.96	40.07	HV(26.05)	*N37D* appears together with *N37E* in patient treated with LPV + RTV [50]	NP/NP
*R41K*	19.00	19.00	SC(2.39)	Associated with reduced in vivo virological responses to RTV + APV in PIs experienced patients [49]	NP
*R57K*	17.88	18.38	*	Relatively frequent in patients failing treatment with RTV + SQV [51]	NP
L63A	3.48	84.77	HV(22.02)	*L63A* frequent polymorphism but significantly associated with the antiretroviral treatment [39,52]	NP
H69Y	3.15	7.62	V(7.56)	Appears in viruses selected with LPV [53]	NP
*K70E*	3.31	9.11	V(7.56)	*K70E* appears in virus selected in cell culture with DRV [54]	NP
*I72R*	1.32	24.50	HV(27.48)	Associated with viral rebound during therapy with LPV + RTV [50]	UM

*p*, prevalence; PV, phenotypic variation; C, conserved; SC, semi-conserved; V; variable; HV, highly variable; APV, amprenavir; ATV, atazanavir; DRV, darunavir; IDV, indinavir; RTV, ritonavir; SQV, saquinavir; PIs, protease inhibitors; NP, natural polymorphisms; UM, unusual mutations.

*Values below the 15th percentile or above the 75th percentile were not considered.

The I13V (17.33%), E35D (18.21%), R41K (19%) and R57K (17.88%) mutations had a *p* ≥ 10% and were located in polymorphic positions observed in non-B subtypes [35,55,56]. In the HIVdb, W6R and I72R are unusual mutations with a frequency <0.05% that only emerge after multiple major and minor resistance mutations [57]. Table 2 shows 41 mutations with a *p >*1% that have not been associated with resistance, 25 are natural polymorphisms and the remaining 16 were unusual mutations. According to phenotypic conservation analysis, the *L5F* and *Q7E* mutations were within the conserved regions, while *D29V*, *P39S, K43R, Q61E, E65D, C67F, P79L, T91V* and *Q92G/K* were within semi-conserved regions. The *T12P/S*, *K14R*, *G17D/E, Q18H, L19I/V/T, G68E, H69Q* and *K70R/T/I* mutations were within the variable regions, and *N37S/T/C/H/I, L63S/V/R/G/H, I72V/T/E/M* and *I93F* were in highly variable regions.

**Table 2:** **Natural polymorphisms and unusual mutations of HIV-1 protease *(p*** **> 1%) without evidence of resistance to PIs**

**Mutation**	***p *** **(%)**	**PV (%)**	**Region (PV)**	**Classification**
*L5F*	1.67	1.67	C (0.95)	UM
*Q7E*	1.52	1.89	C (0.95)	UM
*T12P/S*	4.03/1.34	9.40	V (8.04)	NP/NP
*K14R*	9.60	11.26	V (9.97)	NP
*G17D/E*	1.99/1.32	3.31	V (9.97)	UM/UM
*Q18H*	1.32	3.64	V (9.97)	NP
*L19I/V/T*	4.64/1.32/ 1.32	7.95	V (9.97)	NP/NP/NP
*D29V*	1.32	2.65	SC(1.24)	UM
*N37S/T/C/H/I*	14.4/2.81/1.99/ 1.66/1.32	40.07	HV(26.05)	NP/NP/NP/NP/UM
*P39S*	2.98	4.97	SC(2.09)	NP
*K43R*	3.64	3.97	SC(2.09)	NP
*Q61E*	2.65	3.97	SC(4.47)	NP
*L63S/V/R/G/H*	1.99/149/1.99/1.32/1.32	84.77	HV(22.02)	NP/NP/UM/UM/NP
*E65D*	2.0	2.67	SC(2.0)	NP
*C67F*	2.0	3.33	SC(2.0)	NP
*G68E*	4.30	5.96	V(7.56)	UM
*H69Q*	1.99	7.62	V(7.56)	NP
*K70R/T/I*	1.99/1.32/1.16	9.11	V(7.56)	NP/UM/NP
*I72V/T/E/M*	11.26/6.95/2.32/1.32	24.50	HV(27.48)	NP/NP/NP/UM
*P79L*	1.32	2.48	SC(1.53)	UM
*T91V*	3.33	3.33	SC(2.15)	UM
*Q92G/K*	2.03/2.03	4.05	SC(2.15)	UM/UM
*I93F*	1.35	47.97	HV(47.63)	UM

*p*, prevalence; PV, phenotypic variation; C, Conserved; SC, semi-conserved; V, variable; HV, highly variable; NP, natural polymorphisms; UM, unusual mutations.

Among the codons presented in the Table 2, the mutations in positions K43, L63, H69 and I93 were located in sites associated with minor resistance, but the distance between its localization and the enzyme’s active site reduces the possibility of the structure contributing to drug resistance. All the described mutations could be due to random transcriptional errors, or positive selection from drug and/or immunological stressors [37,58]. Generally, natural polymorphisms occur in remote regions away from the active site, and form domains that define the shape of the homodimer. However, unusual mutations are found in positions associated with drug resistance and possibly generate allosteric changes in the binding site that favour enzymatic function, or decrease the affinity with certain PIs [59]. Therefore, the study of such structural changes produced by these emerging mutations may help in determining the new effects of PIs with different affinities.

**Figure 3:**
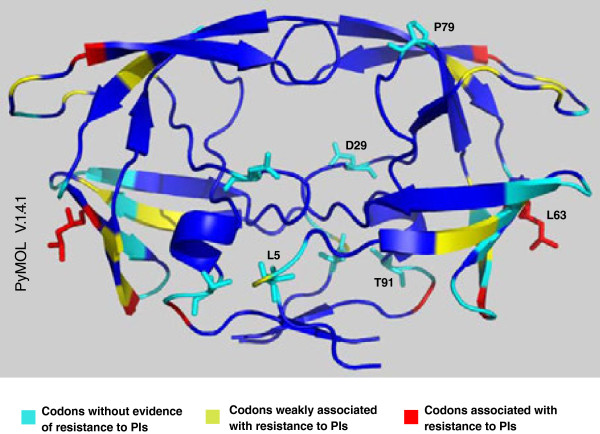
**Codons with natural polymorphisms and unusual mutations in the HIV-1 PR tertiary structure.** Codons in the PR that were not associated with PI resistance (cyan), weakly associated with PI resistance (yellow), and associated with PI resistance (red).

Figure 3 shows PR tertiary structure positions that are: not associated with PI resistance; weakly associated with PI resistance; associated with PI resistance. We have also presented the locations of natural polymorphisms and unusual mutations (Figure 3). The codons T12, N37, L63, H69, K70 and I72 include mutations weakly associated with PI resistance (*T12A/I*, *N37D/E*, *L63A*, *H69Y*, *K70E*, and *I72R*), and mutations lacking evidence of PI resistance (*T12P/S*, *N37S/T/C/H/I*, *L63S/V/R/G/H*, *H69Q*, *K70R/T/I*, and *I72V/T/E/M*).

The *D29V* and *P79L* mutations are located near the active site of the protease, and therefore possibly contribute to the generation of PI resistance. It is of interest to evaluate these unusual mutations *in silico*, and establish their association with resistance to PIs.

### Phenotypic conservation of HIV-1 protease

**Figure 4:**
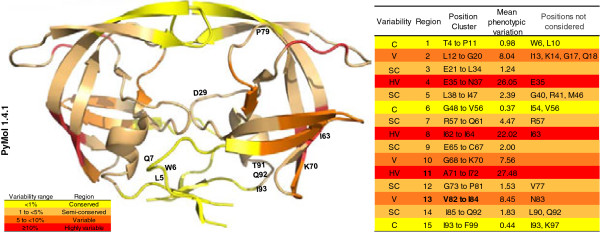
**Phenotypic conservation of HIV-1 PR isolated from Mexican patients.** A consensus sequence was obtained from 151 individual sequences. Regions are shown from red to yellow in proportion to their phenotypic variation (PV,%). Positions with natural polymorphisms and unusual mutations at drug resistance codons are shown in the protease model. Mutations were clustered into 15 regions between codons 4–99 of the protease.

Figure 4 shows the conserved, semi-conserved, variable and highly variable regions of PRs according to PV. Mutations were clustered into 15 regions, for amino acids 4–99 of the protease. For average PV calculation, when the asymmetry in the distribution was greater than 1.4 between the 15th and 75th percentiles, the residues were not considered. We found three conserved, three variable, three highly variable and six semi-conserved regions for each chain. The positions excluded from the PV calculated for each region were W6, L10, I13, K14, G17, Q18, E35, G40, R41, M46, I54, V56, R57, I63, V77, N83, L90, Q92, I93 and K97. The PV in these codons had very different values from those presented by the codons in their respective regions. According to our model of protease conservation, the LES formed by fragments 23–33 and 74–78 were in semi-conserved regions (E21–L34 and G73–P81, except for V77). The LES formed by the 83–92 fragment involved two codons with variable PV, I84 (6.29%) and L90 (12.33%), and two codons with semi-conserved PV, T91 (3.33%) and Q92 (4.05%) [8,60]. Codon 90 contained a drug resistance mutation (*L90M*) common for most PIs, with the exception of DRV and TPV, while T91 and Q92 contained the *T91V*, *Q92G*, and *Q92K* mutations, which are classified in the literature as unusual mutations. The prevalence of the *L90M*, *T91V*, *Q92G*, and *Q92K* mutations was 12.0, 3.33, 2.03 and 2.03%, respectively. Although the effectiveness and specificity of PR proteolytic activity is determined by its active site (amino acids 25–29), these characteristics are influenced by mutations in neighbouring structures, which mainly affect intramolecular interactions with the active site [5,38,42,61]. Contiguous regions and the active site have a semi-conserved state, with a PV of 1.2%. It has been shown that active sites with poor capacity to carry out structural changes help adjust the specificity of natural substrates without losing proteolytic effectiveness [45]. A study that identified the minimal conserved structure of HIV-1 PR, in the presence or absence of drug stress, showed that most of the PV is a product of pharmacological stress [62]. In contrast, the peripheral structural regions have a relatively high PV (for variable and highly variable regions) courtesy of negative selection, and to a lesser extent through resistance of HIV-1 to immune stress [63,64].

### Selective pressure in the *pr* fragment of HIV-1 *pol*

Antiretroviral treatment can exert strong selective pressures within *pol*, which transcribes PR, reverse transcriptase and integrase [62,65]. We have presented the selection pressure results for 10 codons with natural polymorphisms and unusual mutations (Table 3). According to these results, codons 5, 29, 63, 79, 91 and 93 represent positive pressure (dN–dS > 1) through the ML substitution model using the HyPhy algorithm. When these results are compared with the data available in the UCLA HIV Positive Selection Mutation Database, only codons 63 and 93 were consistent with positive selection under immunological and/or pharmacological stressors [26]. The difference in selection pressure for codons 5, 29, 79 and 91 could be due to variability in the antiretroviral regimen sequences administered to Mexican patients. In addition to positive selection, the aligned sites often evolve at different rates because of other biological factors that include site-specific mutation rates and functional constraints of amino acid substitutions [66].

**Table 3:** Selection pressure for codons with unusual mutations and natural polymorphisms

**Codon**	**Triplet**	**PV (%)**	**dN-dS**	**dN-dS (N)**	P value*	**Mutations**
5	CTT	1.67	2.29	0.48	0.285	*L5F*
6	TGG	8.20	−5.97	−1.26	0.996	*W6R*
7	CAA	1.89	−15.57	−3.29	1.000	*Q7E*
29	GAT	2.65	20.20	4.26	0.003	*D29V*
63	CCC	84.77	2.01	0.42	0.196	*L63A/R/S/V/G/H*
70	AAA	9.11	−1.51	−0.32	0.964	*K70I*
79	CCT	2.48	4.44	0.94	0.190	*P79L*
91	ACT	3.33	5.01	1.12	0.086	*T91V*
92	CAG	4.05	−15.62	−3.46	0.990	*Q92G;Q92K*
93	ATT	47.97	28.64	6.34	1.000	*I93F*

*P < 0.05 was considered statistically significant for positive pressure.

PV, phenotypic variation; dN, number of non-synonymous substitutions per site (n/N); dS, number of synonymous substitutions per site (s/S); N, normalized; P, probability.

The codons that were not associated with resistance due to pharmacological stress, and had PV ≥2% were D29 (2.65%) and P79 (2.48%). These were located near the active site of the enzyme; T91 (3.33%) was also found to be necessary for the establishment of the PR dimer. Codons associated with resistance due to pharmacological stress and PV ≥2% were I47 (2.65%), V82 (10.6%) and I84 (6.29%). Only one of these sequences belonged to a naïve individual, with a mutation at V82; the remaining sequences were from treated individuals (three were treated with reverse transcriptase inhibitors only, the remainder were given reverse transcriptase inhibitors and at least one PI).

### Structural prediction of mutant HIV-1 PRs

The molecular structure of all mutant HIV-1 PRs was predicted by comparative homology modelling using the wild-type HIV-1 PR as a template [PDB: 1GNO]. This structure had higher sequence identity compared with the HIV-1 subtype B consensus PR sequence available from the HIVdb. Additional file 1: Table S1 shows the % identity, the expected value of the alignment with the template sequence (E), and the score for the absolute quality of the models. We modelled the proteins with unusual mutations (*L5F, D29V, L63G, L63R, P79L* and *T91V*), natural polymorphisms (*L63H* and*L63S*), and drug-resistant mutant PRs with single mutations or patterns of mutations (*D30N, V32I, M36I, M46I, I47V, G48V, I50V, I50L, I54M, Q58E, T74P, L76V, V82A, V82L, N83D, N88S, I84V,* and *L90M*).

**Figure 5:**
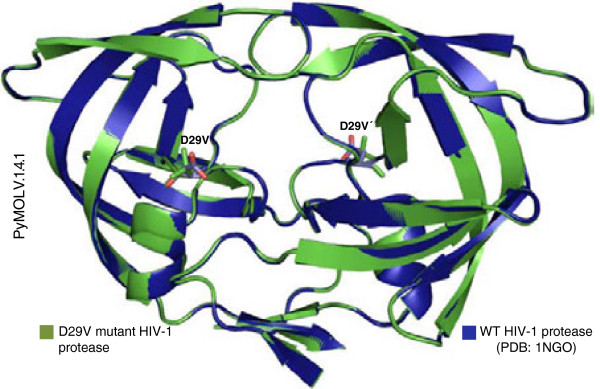
**Wild-type and *D29V *mutant protease structures.** The structure of the wild-type HIV-1 protease (WT) was obtained by X-ray crystallography. [PDB: 1GNO] (blue), and the mutant protease (green) can be clearly seen, with the red structures corresponding to the oxygen atoms of D29.

The model’s accuracy was increased because of the identity between the mutant and template sequences; therefore, we concluded that the model was suitable for all structures. The low E values obtained from the modelled proteins indicate template identification, and adequate target template alignment [27]. The reliability of the predicted structures with natural polymorphisms and unusual mutations in drug resistance positions ranged 0.87–0.89, while positions for major mutation proteins ranged 0.83–0.91. The lower QMEAN4 values correlated with mutants containing patterns of resistance, as a result of the reduced identity of these proteins with respect to the template structure [67]. The QMEAN4 values were acceptable for all the modelled structures. Figure 5 shows the overlapping structures of the wild-type PR [PDB: 1GNO] and the *D29V* mutant, with high similarity between both structures, as well as a difference in the location of the mutation site (position 29).

D29 plays a crucial role for the folding of retroviral PRs [38,68]. Using crystallography, it has been shown that D29 forms hydrogen bonds with R87, which partially constitutes the highly conserved triad, G86 − R87 − D88 [62]. The loss of this specific interaction between α − helix 1 (residues 87–91) and D29 destabilizes the dimer interface [69]. The PR structures of related viruses such as HIV − 2, equine infectious anaemia virus (EIAV), feline immunodeficiency virus (FIV), rous sarcoma virus (RSV) and simian immunodeficiency virus (SIV), also demonstrate a proximity between side chains R87 and D29 [70].

The *in silico* modelling of mutant proteins generated structures very similar to those obtained by X-ray crystallography. The structures with natural polymorphisms, unusual mutations in drug resistance positions, and drug resistance mutations obtained by comparative homology modelling, were appropriate for molecular docking with their respective inhibitors.

### Natural polymorphisms and unusual mutations in PRs and their effects on the free energy of binding by PIs

We have presented the free energy of binding (kcal/mol), as well as the average value of the five lowest energy conformations for the complexes formed by PRs and the main PIs (Table 4). The wild-type PR had the lowest free energy of binding for all PIs, except for IDV, compared with mutant PRs containing major and multiple drug resistance mutations. The magnitudes corresponding to the minor values of the free energy of binding to the reference protein were: wild-type protease-1GNO < major drug-resistant mutant proteases < multiple drug-resistant mutant proteases. The PIs had the greatest degree of affinity for PR 1GNO, consistent with the wild-type PR, whereas reduced affinity for mutant PRs was proportional to the number of mutations [46].

**Table 4:** **Free energy of binding for protease-PI complexes obtained *in silico***

**Inhibitor**	**1GNO-Ligand kcal/mol**	**Mutant protease with major drug resistance mutation kcal/mol**	**Multiple mutant kcal/mol**	**Mutant protease with emergent mutations *L5F/D29V/L63G/L63H/L63R/L63S/P79L/T91V *** **kcal/mol**
Amprenavir	-8,5(-8,4)	-8,4(-7,9)/*I50V* -8,2(-8,12)/*I84V*	-8,0(-7,66)	-8,8(-8,38)/-8,7(-8,28) /-8,8(-8,44)/ -8,8(-8,3)/ -8,8(-8,48)/ -8,7(-8,28)/ -8,8(-8,32)/-8,8(-8,48)
Atazanavir	-8,3(-8,1)	-7,3(-6,94)/*I50L***-8,5(-8,14)/*I84V*** -8,2(-7,98)/*N88S*	-7,1(-6,86)	-7,9(-7,82)/ -8,2(-8,14)/ -8(-7,62)/-8,1(-7,96)/ -8,2(-8,04)/-8,2(-8,06)/-9(-8,8)/-8,2(-8)
Darunavir	-9,3(-8,96)	-9(-8,54)/*I47V* -9,3(-8,86)/*I50V***-9,4(-8,84)/*I54M*** -9,1(-8,86)/ *I84V*	-8,1(-7,9)	-9,4(-9)/ -8,8(-8,56)/-9,4(-8,9)/-9,4(-8,92)/ -9,4(-8,92)/-9,4(-8,88) /-9,4(-8,86)/-9,4(-8,88)
Indinavir	-10,4(-10,02)	**-10,8(-10,5)/*M46I*** -10,8(10,4)/*V82A***-10,5(-10,28)/*I84V***	**-10,7 (-10,26)**	-10,4(-10,18)/-10,1(-9,92)/-10,8(-10,48)/-10,4(-10)/ -10,4(-10,18)/-10,4(-9,8)/-10,4(-10,02)/-10,7(-10,46)
Lopinavir	-10,3(-9,84)	-9,9(-9,56)/*V32I* -9,4(-8,92)/*I47V* -9,5(-8,62)*/L76V* -9,7(-9,24)/*V82A*	-9,7(-9,14)	-9,9(-9,56) /-9,7(-9,18) /-9,6(-9,24)/-9,3(-8,86)/ -9(-8,32)/-9,6(-9)/-9,5(-9,2)/-9,6(-9,42)
Nelfinavir	-10,3(-9,46)	-10(-9,44)/*D30N* -10,1(-9,8)/*L90M*	-9,9(-9,46)	-10,1(-9,62) /-10,4(-9,8)/-10,1(-9,74)/-10(-9,84)/ -10,1(-9,72)/-10,1(-9,74) /-10(-9,78)/-10,1(-9,7)
Saquinavir	-10,9(-10,46)	-10,9(-10,6)/*G48V* -10,4(-10,2)/*L90M*	-10,9 (-10,54)	-9,9(-9,64)/-10,3(-9,62) /-10,4(-10,06)/-10,6(-10,44)/ -10,6(-10,42)/-10,6(-10,42)/ -10,6(-10,5)/-10,5(-10,2)
Tipranavir	-10,6(-10,1)	-10,4(-9,8)/*I47V* -10,3(-10,04)/*Q58E* -10,2(-10,0)/*T74P* -10,4(-9,9)/*V82L* -10,2(-9,6)/*N83D* -10,6(-10,12)/*I84V*	-9,9(-9,64)	-10,2(-9,62) /-10,1(-9,92) /-10,3(-9,98)/-10,2(-9,88)/ -10,2(-9,72)/-10,3(-9,72)/-10,3(-9,72)/-10,2(-9,78)
Ritonavir	-8,0(-7,85)	-7,82(-7,44)/*I47V* -7,8(-7,56)/*I50V* -7,4(-6,94)/*V82L* -7,87(-7,58)/*I84V* -7.9(-7,72)/*L90M*	-7,26(-6,69)	-8,3(-7,93)/-7,85(-7,54)/ -7,75(-7,52)/-7,9(-7,76)/ -8,3(-8,14)/-8,2(-8,09)/-8,4(-8,8)/-7,79(-7,52)

The average value of the five conformations with less free energy of binding of the protease-PI complex is presented in parentheses. Values that do not correspond to decreasing order of free energy of binding are presented in boldface type.

Among the PIs, IDV demonstrated a higher affinity for mutant proteins than PR [PDB: 1GNO]. Additionally, a study that correlated the *in vivo* genetic resistance of HIV-1 to IDV indicated that the development of resistance occurs through the combined effects of several mutations, which do not confer a measurable degree of resistance when they occur on their own [39]. For the other PIs, significant viral resistance has been shown to be a result of the appearance of one or two substitutions in drug-resistance positions [40,71].

The difference between affinities of complexes formed by wild-type and drug-resistant PRs indicates some contribution of phenotypic changes towards PI resistance [72,73]. The complexes with the largest differences involved ATV and DRV, both with a difference of −1.2 kcal/mol. This indicates high susceptibility of both compounds to drug resistance mutations. Lower differences were observed (between −0.7 and −0.3 kcal/mol) for other complexes, indicating these drug resistance mutations have a minor or supplementary effect [72,73].

We obtained a positive value when we calculated the difference of the free energy of binding between the wild-type-IDV complex and the drug-resistant mutant-IDV complex. This is consistent with a high genetic barrier to resistance for IDV, which has lower susceptibility to drug-resistance mutations compared with other PIs [39]. When comparing the free energy of binding between the complexes with drug resistance mutations versus natural polymorphisms and unusual mutation complexes, resistance to ATV, LPV, NFV, and TPV was always observed. The PRs with *L5F*, *D29V*, *L63G*, *L63H*, *L63R*, *L63S*, and *P79L* mutations had lower or equal free energy of binding to ATV, LPV, NFV and TPV, than those with wild-type or drug-resistant PRs.

**Figure 6:**
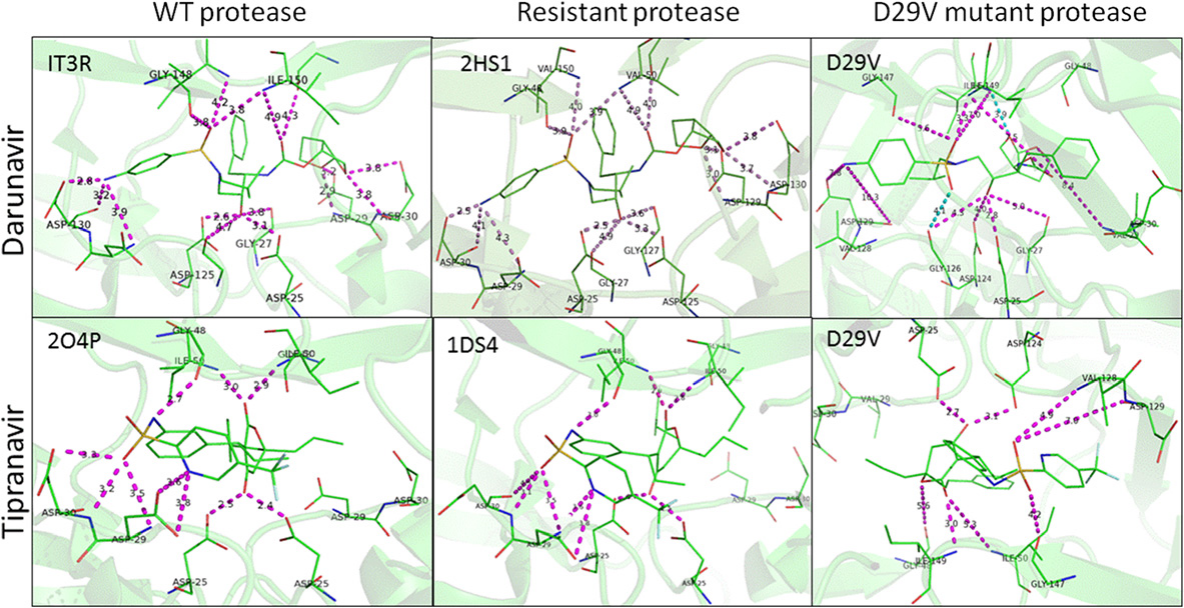
**HIV-1 protease structures.** Wild-type (WT), resistant and *D29V* mutant proteases coupled to darunavir (top) and tipranavir (bottom). The numbers in the left upper corner are the PDB ID numbers used to model darunavir and tipranavir with the protease and to measure the distance (Å) between functional groups (purple).

The complex formed by the *D29V* mutant showed considerable differences between the distance of the V29 and D30 C_α_ -atoms and the heteroatoms closest to the PIs (Table 4). This is probably because of the absence of the C_β_ carboxyl group in the valine compared with the wild-type D29. The electrostatic interactions exercised by the D29 carboxyl oxygens provide stronger affinity to PIs in the active site, resulting in greater affinity compared with the V29 mutant [5,74,75]. The absence of V29 carboxyl oxygens decreases the level of interactions, thus decreasing affinity. Such differences can be observed when measuring the distance between the functional groups of the wild-type, resistant and *D29V* mutant PRs docked to DRV and TPV (Figure 6). For each natural polymorphism and unusual mutation, Table 5 shows the degree of resistance to PIs based on free energy of binding differences when compared with reference PRs.

**Table 5:** Distances (Å) between the amino acid of the protease and the PI heteroatoms

		**Protease**		**aa/Chain-PIs**		**Protease**	
**aa/Chain-PIs**	**Wild type**	**Resistant**	**Emergent**		**Wild type**	**Resistant**	**Emergent**
**PR-IDV**	**[2R5P]**	**[1K6C]**	*D29V*	**PR-LPV**	**[2Q5K]**	**[1RV7]**	*D29V*
29/A-N_2_	6.1	6.1	5.4	25/A-O_4_	5.4	6.8	9.1
29/B-O_4_	3.9	4.2	4.8	25/B-O_4_	5.5	7.8	9.9
30/A-N_2_	6.5	6.6	6.3	29/A-O_1_	5.8	10.8	6
30/B-O_4_	6.3	6.4	7	29/B-O_3_	6	10.3	5.3
82/A-O_2_	10.1	10.7	11.2	30/A-O_1_	3.7	9.8	7.1
82/B-N_1_	6.1	6.2	7.5	30/B-O_3_	6.7	10.1	5.3
84/A-O_2_	8.3	8.2	9.3	84/A-O_4_	8.2	10.1	10.4
84/B-N_1_	7.9	7.9	8.4	84/B-O_3_	7.6	8.1	8.2
**PR-SQV**	**[3OXC]**	**[3CYW]**	*D29V*	**PR-RTV**	**[3NDX]**	**[1RL8]**	*D29V*
29/A-O_D1_	4.1	5.3	3.5	29/A-O_76_	4	3.8	4.9
29/B-N_3_	6.1	6	7.5	29/B-N_5_	5.6	4	5.1
30/A-O_D1_	3.8	4.2	3.8	30/A-O_76_	6.1	6.1	6.9
30/B-N_3_	6.5	6.4	7.5	30/B-S_3_	4	5.5	8.8
48/A-N_D2_	3.4	5.6	7.9	82/A-N_11_	9.4	9.5	9.6
48/B-N_3_	4	6.3	6.3	82/B-O_7_	10.5	10.3	11.6
**PR-APV**	**[3NU3]**	**[3NU4]**	*D29V*	**PR-DRV**	**[IT3R]**	**[2HS1]**	*D29V*
29/A-O_6_	4.5	4.2	4	29/A-O_26_	3.9	4.1	10.4
29/B-N_3_	4.3	4.6	7.6	29/B-N_1_	4.6	4.4	7.3
30/A-O_6_	4.5	3.8	3.8	30/A-O_26_	3.8	3.8	12.3
30/B-N_3_	3.3	3.7	10.7	30/B-N_1_	3.8	3.7	9.5
32/A-O_6_	6.6	6.6	6.9	32/A-O_26_	7.2	7.5	13.9
32/B-N_3_	6.3	6.3	15	32/B-N_1_	6.1	7.6	13.3
**PR-TPV**	**[2O4P]**	**[1DS4]**	*D29V*	**PR-ATV**	**[3EKY]**	**[3OXX]**	*D29V*
29/A-N_28_	6.1	6.2	9.6	29/A-O_AI_	3.9	5.1	5.6
29/B-O_1_	9.1	9.4	8.4	29/B-O_AJ_	3.9	4	8.3
30/A-N_28_	6.2	6	8.7	30/A-O_AI_	6.1	6	8.7
30/B-O_1_	10	10.2	8.6	30/B-O_AJ_	6	6	8.5
82/A-O_8_	10.7	10	10.2	50/A-O_1_	5.7	5.6	4.8
82/B-O_7_	10.3	9.9	7.7	50/B-O_1_	5.3	5.4	3.6
84/A-O_8_	8.3	8.1	8.5				
84/B-O_8_	8.4	7.8	8.1				
**PR-NFV**	**[3EKX]**	**[2PYM]**	*D29V*				
29/A-N_12_	5.9	6.3	5.8				
29/B-O_38_	5	4.4	3.8				
30/A-N_12_	6.4	6.7	6.3				
30/B-O_38_	3.9	3.7	3.7				

The classification of atoms corresponds to the PDB file visualized in PyMOL. Measurements correspond to the distance between the alpha carbon of the PR amino acids and the heteroatoms of the inhibitor, and are expressed as number of amino acids/chain PR − PI heteroatom.

PIs, protease inhibitors; aa, amino acid; PR, protease; APV, amprenavir; ATV, atazanavir; DRV, darunavir; IDV, indinavir; LPV, lopinavir; RTV, ritonavir; SQV, saquinavir; TPV, tipranavir.

Of the emerging mutations, *D29V* appears to favour resistance *in silico* in seven of nine PIs. Designing more effective DRV analogues requires an interaction between D29 and the bis-tetrahydrofuran ring, as this contributes to complex stability [5,42]. All complexes that formed among the PRs with natural polymorphisms, unusual mutations and drug resistance mutations to TPV and LPV had similar free energies of binding. TPV mainly forms hydrogen bonds with residues D25, D29, D30, G48 and I50, while LPV interacts with G27, D29 and D30. A study that elucidated the mechanism by which PIs minimize the harmful effects of resistance mutations, showed that TPV, ATV, LPV, APV, IDV and DRV conserve their antiretroviral activity in the presence of drug resistance mutations. This phenomenon is due to the compensation of the loss of enthalpy (ΔH) with an entropy gain (−TΔS), except in the case of TPV [75]. Our results are consistent with another report that showed isolated strains with a high level of phenotypic resistance to LPV were susceptible to other PIs [76]. This corresponds with PR resistance to TPV and LPV that contain emerging mutations whose free energies of binding were greater than those obtained with wild-type PR.

We found a high prevalence (89%) of *L63PGHRS* mutations in HIV-1 variants isolated from Mexican individuals, probably because of the prevalence of HIV-1 subtype B [32,33]. In the present study, among the functional groups found at position 63 (*L63G*, *L63S*, *L63H* and *L63R*), only glycine had hydrophobic characteristics, while serine was hydrophilic, and histidine and arginine were alkaline. These four mutations conferred resistance to NFV, ATV, TPV and LPV, most probably through an allosteric effect, given that the substitutions were not located close to the residues where the PIs bind [10,74]. Few mutations at position 63 have been examined for their resistance effects to PIs. The *L63P* mutation has a compensatory effect that increases catalytic activity from 110% to 530%; when *L63P* is associated with *M46I*, it forms a combination that is resistant to APV, IDV, LPV or NFV [9,77]. Residue 63 provides hydrophobic contacts between the slit of the loop formed by amino acids 38–42 and a β-sheet (residues 59–63) [74]. Although the study of mutations in this position has been limited to *L63P* to assess the effect of mutations that provide non-hydrophobic characteristics, alternative mechanisms could be shown by which HIV-1 PR compensates for pharmacological stressors.

### Clinical characteristics of patients with unusual mutations at resistance sites and/or natural polymorphisms

Of the participating individuals, 48 of 151 (31.8%) showed resistance to at least one PI. Of these, 34 (70.8%) showed a high level of resistance, four (8.3%) showed intermediate levels of resistance, and 10 (20.8%) showed low level resistance.

Of the 151 sequences, 24 (15.9%) had one or more unusual mutations at resistance sites and/or natural polymorphisms. They were isolated from 21 (87.5%) male and three (12.5%) female patients; 70.8% of whom lived in the central-east of Mexico, and 29.2% in the north-west. Of these 24 patients, 23 (95.8%) received antiretroviral therapy, and one (4.2%) was naïve to treatment. The nucleoside reverse transcriptase inhibitors (NRTIs) and non-nucleoside reverse transcriptase inhibitors (NNRTIs) used, in order of frequency, were AZT, 3TC, ddI, d4T, ddC, NVP, EFV and ABC. The main PIs used were IDV, RTV and SQV. The average viral load in this group of patients was 228,225 virus copies/mL and a mean CD4^+^ lymphocyte count of 223 cells/μL. According to the case definition of HIV infection and AIDS by the Centers for Disease Control and Prevention (Atlanta, USA) [78], patients were classified as asymptomatic (*n* = 2, 8.5%), symptomatic (*n* = 6, 25%), AIDS (*n* = 12, 50%), and of unknown clinical category (*n* = 4, 16.5%).

## Conclusions

The use of bioinformatics to identify potential mutations that confer resistance to antiretroviral drugs allows researchers to develop realistic three-dimensional models that illustrate the atomic interactions between an enzyme and its substrate. *In silico,* the structural correlation of natural polymorphisms and unusual mutations of drug resistance codons, allows the identification of HIV-1 variants resistant to PIs. The *D29V* mutation increases the probability of resistance to PIs as it generates unstable complexes at the HIV-1 protease active site. The prevalence of this mutation in different populations should be further studied, and parallel crystallographic studies are required to confirm our *in silico* findings.

Among mutant PRs-PIs complexes evaluated, TPV and LPV had free energies of binding greater than those obtained with wild-type PRs.

Furthermore, the presence of a high rate of *L63P*, *I93L*, *V77I* and *I62V* polymorphisms among the Mexican population is similar to that observed in patients that underwent antiretroviral treatments in other American and western European countries. These data reinforced the knowledge regarding the molecular epidemiology of the HIV-1 subtype B in Mexico through the presence of HIV polymorphisms.

## Endnote

The Contents of this publication are the authors responsibility and do not necessarily represent the official views of the Instituto Mexicano del Seguro Social.

## Abbreviations

FDA: USA Food and Drug Administration; HAART: Highly Active Antiretroviral Therapy; HIV: Human Immunodeficiency Virus; LES: Local Elementary Structures; MEGA: Molecular Evolution Genetics Analysis; PDB: Protein Date Bank; PIs: Protease Inhibitors; PV: Phenotypic Variation; QMEAN: Qualitative Model Energy Analysis; PR: protease; LANL: Los Alamos HIV sequence Database; HIVDB: HIV Drug Resistance Database; p: prevalence; M: Mutation; ML: Maximun Likelihood; dS: synonimous site divergence; dN: nonsynonimous site divergence.

## Competing interests

The authors declare that they have no competing interest.

## Authors’ contributions

CMM, MED, MVT and MFS performed sequences and bioinformatics analyses. EVV designed the study. AVO, FGG, MCM and LGFR provided clinical samples and collected the data. BTM performed the data analysis, CMM and EVV wrote the manuscript. All authors read and approved the final manuscript.

## Supplementary Material

Additional file 1: Table S1 Identity value, expected value and QMEAN analyses of mutant proteases models tested to estimate the quality of the predicted structure. Click here for file
