# Supplementary material for: Natural polymorphisms and unusual mutations in HIV-1 protease with potential antiretroviral resistance: a bioinformatic analysis
Source: BMC Bioinformatics. 2014 Mar 15;15:72. doi: 10.1186/1471-2105-15-72 (PMC4003850; doi:10.1186/1471-2105-15-72)
Supplement: Additional file 1: Table S1 — Identity value, expected value and QMEAN analyses of mutant proteases models tested to estimate the quality of the predicted structure. [file 1471-2105-15-72-S1.docx]

**Additional File 1** Identity value, expected value and QMEAN analyses of mutant proteases models tested to estimate the quality of the predicted structure

| **Mutant** | **Identity value (%)** | **E value** | **QMEAN4** |
| --- | --- | --- | --- |
| **L5F** | 98.99 | 3.04e-48 | 0.88 |
| **D29V** | 98.98 | 5.23e-47 | 0.84 |
| **L63G** | 98.99 | 2.7e-47 | 0.88 |
| **L63H** | 98.97 | 5.68e-48 | 0.88 |
| **L63R** | 98.99 | 8.62e-48 | 0.89 |
| **L63S** | 98.98 | 7.61e-48 | 0.89 |
| **P79L** | 98.98 | 3.5e-47 | 0.87 |
| **T91V** | 98.97 | 9.45e-48 | 0.88 |
| **I93F** | 98.99 | 2.77e-48 | 0.89 |
| **D30N** | 98.98 | 9.29e-48 | 0.91 |
| **V32I** | 98.94 | 5.59e-48 | 0.88 |
| **M36I** | 98.98 | 1.3e-47 | 0.88 |
| **M46I** | 98.97 | 1.31e-47 | 0.89 |
| **I47V** | 98.98 | 3.44e-48 | 0.89 |
| **G48V** | 98.99 | 2.04e-47 | 0.89 |
| **I50V** | 98.98 | 3.44e-48 | 0.89 |
| **I50L** | 98.97 | 4.3e-47 | 0.88 |
| **I54M** | 98.98 | 4.69e-48 | 0.88 |
| **Q58E** | 98.95 | 4.97e-48 | 0.88 |
| **T74P** | 98.97 | 1.41e-47 | 0.85 |
| **L76V** | 98.99 | 6.07e-48 | 0.89 |
| **V82A** | 98.99 | 5.14e-48 | 0.89 |
| **V82L** | 98.97 | 3.15e-48 | 0.87 |
| **N83D** | 98.99 | 8.0e-43 | 0.89 |
| **N88S** | 98.88 | 1.01e-47 | 0.89 |
| **I84V** | 98.98 | 3.44e-48 | 0.88 |
| **L90M** | 98.99 | 4.21e-48 | 0.87 |
| **I50V,I84V** | 97.98 | 3.39e-48 | 0.88 |
| **I50L,I84V,N88S** | 96.97 | 1.83e-47 | 0.90 |
| **I47V,I50V,I54M, L76V** | 94.9 | 1.18e-46 | 0.88 |
| **M46I,V82A,I84V** | 96.97 | 2.41e-47 | 0.90 |
| **V32I,I47V,L76V, V82A** | 95.96 | 1.89e-47 | 0.90 |
| **D30N,L90M** | 97.98 | 1.33e-47 | 0.88 |
| **G48V,L90M** | 97.98 | 2.73e-47 | 0.88 |
| **I47V,Q58E,T74P,V82L,N83D,I84V** | 93.88 | 1.68e-45 | 0.83 |
| **GU382812** | 90.91 | 7.15e-44 | 0.87 |

*Identity value was calculated as percentage of structural homology between mutant protein and template protein. E value, Expect value; QMEAN, Qualitative Model Energy Analysis.
